# Comparison of effects of P-coumaric acid and coumarin on colorectal cancer cell line by inducing apoptosis and autophagy 

**DOI:** 10.22038/AJP.2024.24194

**Published:** 2024

**Authors:** Elham Hoveizi, Kiavash Hushmandi

**Affiliations:** 1 *Department of Biology, Faculty of Science, Shahid Chamran University of Ahvaz, Ahvaz, Iran*; 2 *Faculty of Veterinary Medicine, Shahid Chamran University of Ahvaz, Ahvaz, Iran *

**Keywords:** Autophagy, Cancer phytotherapy, Coumarin, Signaling pathway

## Abstract

**Objective:**

Autophagy, as a cellular pathway involved in removing damaged proteins and organelles, performs a vital function in the homeostasis and fate of cells. Natural compounds of coumarin (CO) are found in a variety of herbs. Due to their many medicinal properties, including antitumor and anti-proliferative activity, they are involved in apoptosis and autophagy processes. This investigation desired to analyze the apoptotic and autophagic effects of p-coumaric acid (PCA) and CO on HT-29 cells cultured in fibrin hydrogel.

**Materials and Methods:**

Cell viability and apoptotic and autophagic changes were evaluated by MTT assay, Acridine Orange, 4′,6-diamidino-2-phenylindole (DAPI), and monodansylcadaverine (MDC) staining. The expression *Bax*, *Bad*, *Bcl2*, *Lc3*, *Beclin-1*, *P53* and *Atg5* was respectively measured by qRT-PCR and Western blotting.

**Results:**

CO (IC50=25 μM) and PCA (IC50=150 μM) had a dose- and time-dependent cytotoxic effect in HT-29 cells. So, the cytotoxic effects of CO were significantly higher than PCA and these differences were also evident in cell morphology investigations. The data illustrated a high expression of pro-apoptotic and pro-autophagic genes and a declined expression of anti-apoptotic and anti-autophagic genes.

**Conclusion:**

CO (that was more potent) and p-coumaric acid-induced autophagy via PI3K/Akt/mTOR and AMPK/mTOR signaling on HT-29 cells.

## Introduction

Cancer is a prevalent malignant disorder worldwide. Colorectal cancer is one of the most prevalent cancers. With the increasing number of elderly people in non-industrialized countries, the number of people with colon cancer is also increasing rapidly. Cancer is a controversial disease, which has diverse epidemiological factors at the global level (Sitarz et al., 2018 ▶). Genetic and environmental factors such as poor diet, obesity, smoking, and alcohol can be mentioned as factors that promote colon cancer (Karavasiloglou et al., 2019 ▶; Van Blarigan et al., 2018 ▶). Irrepressible cell divisions and escape from apoptosis are considered the initiating factors of this type of cancer (Mi et al., 2017 ▶). Inhibiting DNA synthesis, controlling the production of free radicals, and inducing cell death including apoptosis or autophagy are effective factors in cancer treatment (Gao and Hu, 2010 ▶). Apoptosis is an effective strategy for cell death that happens in reaction to diverse stressors, such as physiological, pathological, or cytotoxic impulses in the body. Apoptosis is indicated by morphological changes, including cell shrinkage, and chromatin condensation (Slattery et al., 2018 ▶). According to the results of previous researches, it can be concluded that the induction of apoptosis process is one of the most useful methods for the treatment of cancers (Liu et al., 2019 ▶). One of the interesting topics in the field of cell death research is devoted to autophagy. Autophagy is an intracellular procedure, which can substantially influence cellular injury by exterior stimulants such as chemical mechanisms, oxidative stress, healthy shortages, and cell protection in stressful situations (Ashrafizadeh et al., 2019 ▶; Wang et al., 2018 ▶). This procedure has a vital function in life, growth, differentiation, and homeostasis and it is concerned with an expansive scope of disorders, including malignant and other diseases (Chun et al., 2014 ▶; Mohammadinejad et al., 2019 ▶). Consequently, multiple medications in the area of cancer therapy handle autophagy. Of course, autophagy has a dual role in cellular processes and it can improve viability by interfering with cell death and may also be associated with apoptosis and cause cell death (Fesik, 2005 ▶). Previous investigations have demonstrated that coumarin (CO) mixtures can perform an important function in controlling cell extinction (Lin et al., 2019 ▶). CO is a natural cyclic compound belonging to the family of benzopyrones. It is consisting of benzene and pyrone rings, which are employed in an assortment of healing applications, including the anti-plasmodial cure of malaria, anti-thrombotic (Goel et al., 2007 ▶), anti-cancer and antibacterial activities (Sarker and Nahar, 2017 ▶), and as a potent antioxidant (Basnet and Skalko-Basnet, 2011 ▶). Previous investigations have revealed that CO and its products have been utilized in the therapy of different cancers, including malignant melanoma (Jeon et al., 2015 ▶), renal cell carcinoma (Musa et al., 2018 ▶), and prostate cancer. Liu et al. prepared a blend combination of CO and phenyl sulfonyl that had significant anti-proliferative activity against lung adenocarcinoma (A549) (Liu et al., 2014 ▶). P-coumaric acid (PCA) is also one of the isomers of coumaric acid (a derivative of cinnamic acid), which is the most common isomer in nature. Research has found that this compound has many medicinal properties, including cardioprotective, anti-melanogenic, and autophagic features (An et al., 2010 ▶; Prasanna et al., 2013 ▶; Shailasree et al., 2015 ▶). PCA interferes with the proliferation of colon cancer cells by stimulating the apoptotic pathway (Jaganathan and Mandal, 2009 ▶). PCA is widely found in fruits, vegetables, and even honey. It has antioxidant possessions that downsize the danger of gastric carcinoma by stopping carcinogenic nitrosamines (Ferguson et al., 2005 ▶). It also has anti-tumor and anti-mutagenic activity (Tan et al., 2015 ▶). 

Earlier examinations directed us to research the anti-cancer impacts and the molecular mechanisms implicated in the induction of apoptosis and autophagy by PCA and CO in HT-29 cells in 3D culture.

## Materials and Methods

**Materials and reagents**

This study was an *in vitro* study performed on human HT-29 cells in the Cell Culture Research Laboratory of the Faculty of Biology. For the preparation of 5, 10, 15, 20, and 25 μM concentrations of CO (Sigma, USA) and 10, 50, 100, 150, and 200 μM concentrations of PCA (Sigma, USA), they were liquefied in the Dulbecco's Modified Eagle Medium (DMEM) media and then purified using a needle filter (Bio Fact BSS20-PE2-, Korea) at 4°C (Musa et al., 2018 ▶).

**Cell culture**

HT-29 cells were prepared from the Pasteur Institute of Tehran and then maintained in DMEM media (Gibco, USA) including 10% FBS (Fetal bovine serum, Gibco, USA) and incubated at 5% CO_2_ and 37℃. The cells were passaged by trypsin/EDTA (0.25%, Gibco, USA) following for density above 80%.

**Fibrin gel scaffold**

For cell culture in the fibrin hydrogel scaffold, M199 medium (Gibco, USA) including 10% FBS, 1% Penicillin/Streptomycin (Pen/Strep) (Gibco, USA), and fibrinogen powder (Sigma, USA) at a dose of 3 mg/ml was used. The fibrin scaffold (M199 medium containing FBS, Pen-Strep, and fibrinogen) initially was added to each well. Then, the cell suspension with the number of 5×10^4^ cells was added and gently aspirated to prevent bubble formation. Subsequently, 15 µl of thrombin was spilled to wells at the dose of 120 µg/ml and immediately gently aspirated until gelatinized. Then the culture dish was incubated. After 2 hr of cell incubation, 500 µl of M199 medium containing 10% FBS serum and 1% antibiotic was added to each well (on the hydrogel) and transferred to the incubator again (**Bayat et al., 2018**). 

**Scanning electron microscopy (SEM) analysis**

For SEM, HT-29 cells were seeded on a fibrin hydrogel scaffold at a density of 5×10^4^ cells for 3 days. Approximately 500 µl of 2.5% glutaraldehyde solution was then added to each well and washed twice with phosphate-buffered saline (PBS) after storage at room temperature for 2 hr. The dehydration steps were performed using alcohol doses (30, 50, 70, 80, 90, and 100%). Then cell-containing scaffolds were freeze-dried and covered with gold particles and imaged by electron microscopy (SEM, Leo 1455VP) (Bayat et al., 2018). 

**Cell viability ass**

Cell viability and IC50 concentrations were estimated by MTT assay. 10×10^3 ^cells/well was seeded on a culture plate and cultured for 24 hr. Then the cells were treated with 5, 10, 15, 20, and 25 μM of CO and 10, 50, 100, 150, and 200 μM of PCA, and cell viability was estimated on days 1, 3, and 5. A control sample was regarded for each test that stayed untreated. Then at the given times, the media was withdrawn from cell-containing wells and 100 ml of the media containing 10 μM of MTT solution (5 mg/ml concentration) was spilled, and the cells were maintained for 3 hr. Then the supernatant was removed and 100 μM of dimethyl sulfoxide (DMSO) (Merck, USA, 100%) was spilled into wells. Optical absorption was estimated at 570 nm using an ELISA (Stat fax 2100, Florida, USA) (Hoveizi et al., 2019 ▶).

**Microscopy and photography**

For the morphological evaluation of HT-29 cells, a suitable camera that was linked to an inverted microscope (Olympus, Japan) was used and the morphology of cells was compared between the control and treatment groups.

**DAPI staining**

To evaluate changes in cell nuclei, HT-29 cells were maintained in a media containing an IC50 concentration of CO and PCA for 24 hr. Then cell supernatants were withdrawn and the cells were rinsed with PBS. The cells were dyed with DAPI (Sigma, USA) with a dose of 1 μg/ml (Hoveizi et al., 2019 ▶). 

**Acridine orange staining**

Acridine orange/ethidium bromide (AO/EB) staining is one of the common staining methods to check cell survival. So, in this double staining, live cells are seen in green and dead cells are seen in orange. For AO/EB (Sigma, USA) staining, HT-29 cells were cultured with IC50 dose of CO and PCA for 24 hr. To carry out the staining, 1 mg/ml AO dye and 1 mg/ml EB dye were set and blended in equivalent ratios. The cells were then died with this solution for 5 min and observed under a fluorescent microscope (FM) (Hoveizi et al., 2019 ▶).

**MDC staining**

In this method, the dye was prepared in 50 µM concentration in PBS then was incubated for 45 min to 1 hr on cells that were treated for 24 hr. After this, the cells were rinsed 3 times with PBS and followed using a FM (Olympus, Japan) with a blue filter (Ashrafizadeh et al., 2019 ▶).

**RNA extraction and qRT-PCR**

For RNA isolation, Trizol-lysed cell extracts were centrifuged twice in chloroform for 13 min at 13,000 rpm and rinsed with isopropanol and 70% alcohol. After drying, RNA was dissolved in diethyl pyrocarbonate (DEPC) water. A nanodrop was used to determine the quantity and quality of RNA. cDNA was synthesized utilizing Taq Man Reverse Transcription Kit (QIAGEN, Japan). In the following, for each sample, the synthesized cDNA (40 ng) was blended with SYBER green master mix (QIAGEN, Japan). Also, primers with a concentration of 10 µg/ml (Table 1) were used for qRT-PCR. CT data investigations were done using StepOne software and standardized by *GAPDH* (as an internal control) gene. The fold change in *Bcl-2*, *Bax*, *p53*, *Atg5*, *Beclin-1*, *Lc3*, and *Bad* expression analogized with the *GAPDH* gene in control examples. The primers were picked from the NCBI site (Table 1) (Hoveizi et al., 2019 ▶).

**Table 1:** Primers and the reaction conditions for qRT-PCR

**Tm (C)**	**Primer Sequence (5'→3')**	
	GCTGGACATTGGACTTCCTC	*BAX* (F)
58/5	ACCACTGTGACCTGCTCCA	*BAX* (R)
	CGGAGGATGAGTGACGAGTT	*BAD* (F)
58/3	CCACCAGGACTGGAAGACTC	*BAD* (R)
	GATGGGATCGTTGCCTTATGC	*BCL-2* (F)
58/8	CCTTGGCATGAGATGCAGGA	*BCL-2* (R)
59/35	GAGTTGCTGACTGACCCTCC GCTCGATGATCACCGGGATTT	*Lc3*(F) *Lc3*(R)
60/1	CTGAAACTGGACACGCTTC	*Beclin-1*(F)
	TGTGGTAAGTAATGGAGCTGTGA	*Beclin-1*(R)
	GGAGGGGCGATAAATACC	*P53*(F)
57/25	AACTGTAACTCCTCAGGCAGGC	*P53*(R)
57/8	GGAGAGAGGAGCCAG TGTTGCCTCCACTGAACT	*Atg5*(F) *Atg5*(R)
58/1	GCAAGAGCACAAGAGGAAGA ACTGTGAGGAGGGGAGATTC	*GAPDH*(F) *GAPDH*(R)

**Western blotting**

Adioimmunoprecipitation assay (RIPA) buffer was used to isolate the protein by protein lysis, and the protein dose was estimated with a bicinchoninic acid (BCA) protein assay kit (Beyotime). Then, whole protein was obtained by Sodium dodecyl-sulfate (SDS)-polyacrylamide gel electrophoresis and transferred to polyvinylidene fluoride (PVDF) membranes. Samples were intercepted with 3% bovine serum albumin including Tween-20 and 0.05% Tris-buffered saline. Membrane was incubated with anti-p-AKT (1/500, Abcam), anti-AKT (1/500, Abcam), anti-p-mTOR (1/1000, Abcam), anti-mTOR (1/2000, Abcam), Anti-p-AMPK (1/500, Abcam), Anti-AMPK (1/500, Abcam), Anti-LC3Ι, Π (1/500, Abcam), Anti-Beclin-1 (1/500, Abcam), Anti-Atg5 (1/1000, Abcam), and anti-GADPH (1/500, Abcam) with a secondary anti-rabbit IgG-HRP antibody (1/1000, Abcam) for 1.5 hr at the room temperature. In the end, the strips were labeled with DAB (Sigma, Germany). Also, protein presentation was analyzed utilizing Image J software (Ashrafizadeh et al., 2019 ▶).

**Statistical analysis**

Statistical analysis was carried out utilizing SPSS software (ANOVA). Diagrams were drawn utilizing Microsoft Excel 2016 software and differences were regarded as significant at the status of p<0.05.

## Results

**Morphological evaluation of fibrin hydrogel scaffolds and cultured cells by scanning electron microscopy**

**Figure 1:**
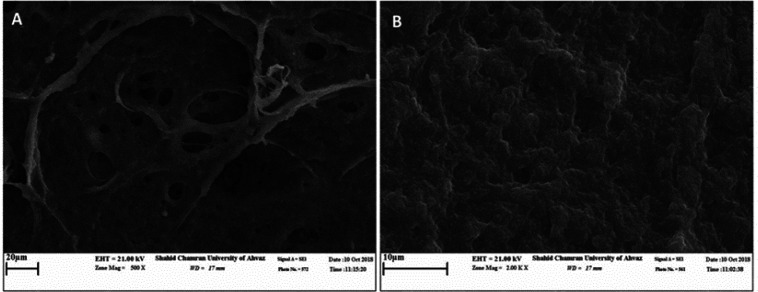
Scanning microscopy results obtained from morphology analysis of fibrin hydrogel scaffold and HT-29 cells cultured after 72 hr. A: cell-free fibrin hydrogel scaffold with the magnification of 1.00 KX. B: HT-29 cells cultured on fibrin hydrogel scaffold with the magnification of 1.00 KX - These images show the desired properties and appropriate porosity of the scaffold

The results of morphological analysis of fibrin hydrogel scaffolds and HT-29 cells cultured in this gel using SEM imaging technique indicated several aspects. These results showed the desired mechanical properties, appropriate porosity of the scaffold, and the appearance and integrity of the scaffolds. Furthermore, the study of cell morphology confirmed the proper binding, adhesion, and association between the cells and the scaffold, preserving proliferation and viability, establishing normal morphology, and maintaining healthy cells (Figure 1).

**Results of MTT assay for cell viability**

**Figure 2:**
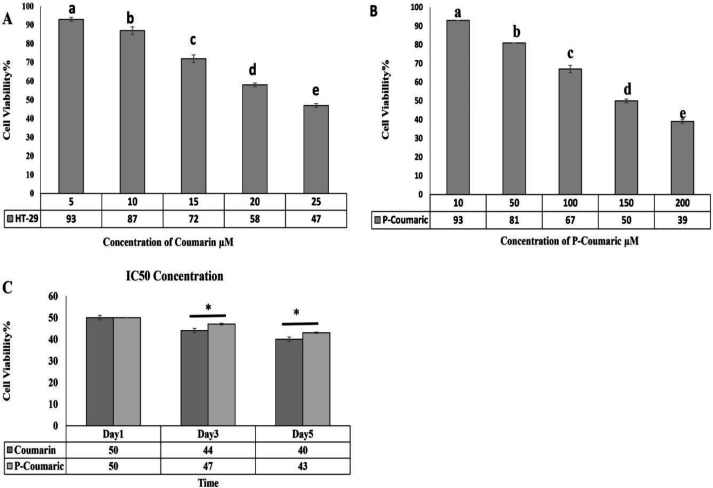
Cell viability charts using MTT assay. A: Effects of different concentrations of coumarin on the viability rate of HT-29 cells on days 1,3, and 5 using MTT assay and determination of 25 μM concentration as IC50 after 24 hr. B: Effects of different concentrations of p-coumaric acid on the viability rate of HT-29 cells on days 1, 3, and 5 using MTT assay and determination of 150 μM concentration as IC50 after 4 hr (n=3). *Significant difference with the control group at p<0.05. Dissimilar letters indicate significant differences

The IC50 of CO was determined to be 25, 20, and 17 μM on days 1, 3, and 5, respectively, for treated HT-29 cells. The outcomes demonstrated that the toxic impacts of this compound on cell viability were time- and dose-dependent and cell viability declined with the growing concentration of CO (p<0.05). Cell viability results (p≤0.05) are shown in Figure 2A. Also, IC50 was determined to be 150, 125, and 100 µM for the PCA for treated HT-29 cells on days 1, 3, and 5 respectively. Cell viability based on MTT assay in cells exposed to 10, 50, 100, 150, and 200 μM concentrations of PCA for HT-29 cells is shown in Figure 2B. According to the MTT assay, cell death significantly developed in HT-29 cells with the growing quantity of PCA (p<0.05). Cell viability of the groups was seriously reduced on day 5 compared to on day 3 and day 1. Additionally, 3-day-treated cells demonstrated a lower viability rate than 1-day-treated cells suggesting that the effects of these compounds are time-dependent. On the other hand, the group treated with CO demonstrated a notable reduction in cell viability on days 1, 3, and 5 compared to PCA (p≤0.05) (Figure 2).

**Morphology of HT-29 cells**

**Figure 3:**
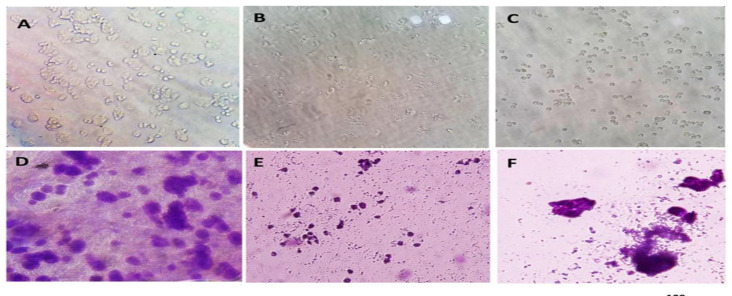
. Morphological study using Giemsa staining of HT-29 cells by invert microscope A: HT-29 control cells B: HT-29 treated cells with IC50 concentration of coumarin after 24 hr C: Treated HT-29 cells with IC50 concentration of p-coumaric acid after 24 hours D: Normal HT-29 cells with Giemsa E: HT-29 cells treated with IC50 concentration of coumarin after 24 hr with Giemsa F: HT-29 cells treated with IC50 concentration of p-coumaric acid after 24 hr by Giemsa staining. In coumarin-treated and p-coumaric acid-treated cells showed a decrease in cell volume and number compaction compared to the control group, confirming cell death

HT-29 control and cells treated with IC50 concentration of CO and PCA were stained with Giemsa staining and examined by invert microscopy. Here, a significant reduction in cell proliferation rate, loss of cell adhesion, and cell shrinkage confirmed cell death in the cells treated with CO and PCA. According to the results, morphological differences were more apparent in cells exposed to CO than PCA (Figure 3A-F).

**Morphological changes of HT-29 cell nuclei**

**Figure 4:**
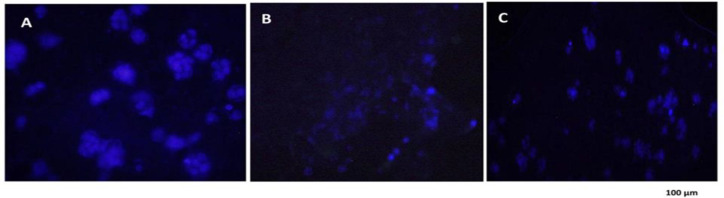
Morphological characterization of the nucleus using DAPI staining in HT-29 cells cultured on fibrin hydrogel scaffold in control group. A: untreated or control, B: treated with 25 μM coumarin, C: treated with 150 μM p-coumaric acid

Following the treatment with IC50 concentration of CO and PCA the cells were stained using DAPI dye and analyzed by fluorescent microscope with violet filter. The nucleus of control cells was spherical, crystal clear, and integrated, indicating good cellular health. But nuclei of cells treated with CO and PCA were found to be isolated, compact, and shrunk. The study of different and random images showed a significant decrease in the number of treated cells compared to the untreated cells (Figure 4). Figure 3H illustrates less dense and shrunk nuclei, indicating better CO performance than PCA.

**Acridine orange/ethidium bromide staining**

**Figure 5:**
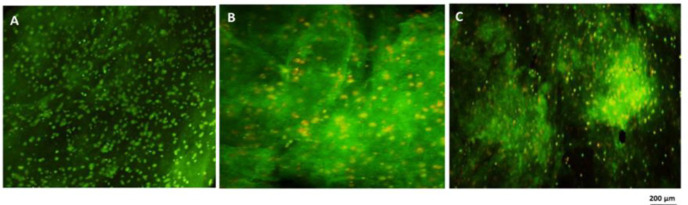
Apoptosis study using acridine orange/ethidium bromide staining of HT-29 cells cultured in fibrin hydrogel scaffold by magnification (20 X) by fluorescent microscopy. A: HT-29 control cells B: HT-29 cells treated with IC50 concentration of coumarin after 24 hr C: HT-29 cells treated with IC50 concentration of p-coumaric acid after 24 hr. Orange-colored cells confirm cell apoptosis and green-colored cells indicate live cells in the samples

HT-29 cells were died with AO/EB after exposure to IC50 dose of CO and PCA. As demonstrated in Figure 5, the control cells were round, clear, and green, due to the entry of acridine dye from the natural cell membrane, indicating their viability and lack of apoptosis. The cells that were exposed to CO and PCA exhibited an orange color and a wrinkled appearance, indicating confirmation of apoptosis and cell death. Based on a random assessment of different parts of different images, it was observed that treated HT-29 cells were more likely to undergo apoptosis and cell death. These cells also exhibited a higher number of orange-colored cells (Figure 5).

**Detection of autophagic vacuoles by monodansylcadaverine** **staining**

**Figure 6:**
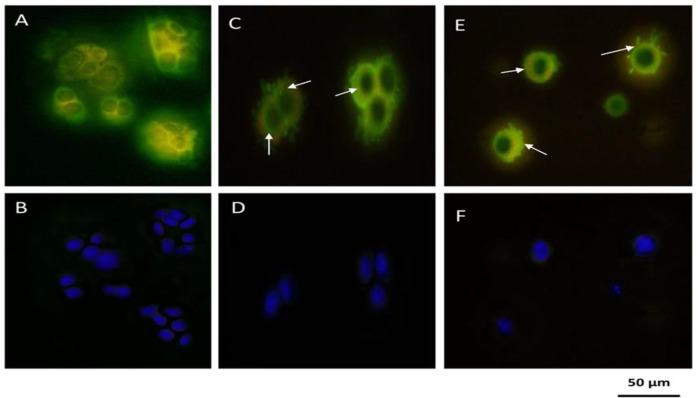
Autophagy study using MDC staining in HT-29 cells cultured in fibrin hydrogel scaffold by fluorescence microscopy. A: MDC staining in control group of HT-29 B: DAPI staining in control group of HT-29 C: MDC staining of cells treated with coumarin at IC50 concentration 24 hr after treatment D: DAPI staining of cells treated with coumarin at IC50 concentration 24 hr after treatment E: MDC staining of cells treated with p-coumaric acid at IC50 concentration 24 hr after treatment F: DAPI staining of cells treated with p-coumaric acid at IC50 concentration 24 hr after treatment

MDC fluorescent staining was used as a marker of autophagy to define the kind of cell dying, morphology, and the presence of autophagic vacuoles. MDC can penetrate from autophagosome lipid membranes into autophagic vacuoles. The autophagic vacuoles of HT-29 cells after exposure to IC50 dose of CO and PCA were observed by fluorescent microscope with a blue filter, to be further evaluated. The presence of autophagic vacuoles in the cytoplasm of the cells and the increased fluorescence intensity of the cells confirmed autophagy in the treated cells (Figure 6).

**qRT-PCR results of autophagic and apoptosis**

**Figure 7:**
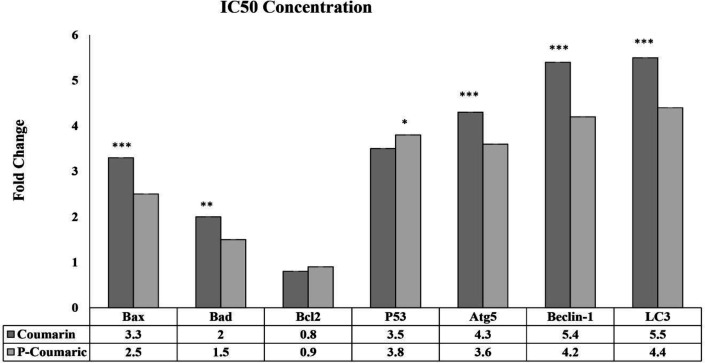
Gene expression alterations in HT-29 cells treated with coumarin and p-coumaric acid. Increased expression of *Bad*, *Bax*, *P53*, *Lc3* genes and decreased expression of *Bcl2* gene in HT-29 cells treated with coumarin and p-coumaric acid A: after 24 hr and B: after 48 hr, confirmed apoptosis and increased expression of *Beclin-1* and *Atg5* genes, approved autophagy. These changes were significant compared to the control group. *** Indicates significant difference with the control group at p<0.001, ** Indicates significant difference with the control group at level (p≤ 0.01), * Significant difference with the control group at level (p≤ 0.01). All experiments were conducted in triplicate, and GAPDH was used as the internal normalizer for gene expression analysis. Also, the control group is considered as the reference with a value of 1.

The results of the study indicated that the expression levels of *Bax*, *P53*, *Bad*, *Atg5*, *Beclin-1*, and *Lc3* genes increased, while the expression of the *Bcl2* gene decreased in HT-29 cells after 24 and 48 hr of treatment with IC50 concentration of CO and PCA. These changes were significantly different compared to the untreated group (p≤0.001), suggesting a diverse impact of the treatment on gene expression in the HT-29 cells. The observed difference in gene expression levels indicates a concurrent activation of apoptosis and autophagy-induced cell death in HT-29 cells when exposed to these compounds. The level of gene expression showed a time-dependent pattern, with significantly higher expression observed at 48 hr compared to 24 hr. Figure 7 presents that the aforesaid genes have extremely risen in CO treated HT-29 cells more than those treated with PCA confirming the greater influence of CO (Figure 7).

**Western blotting results**

**Figure 8 A:**
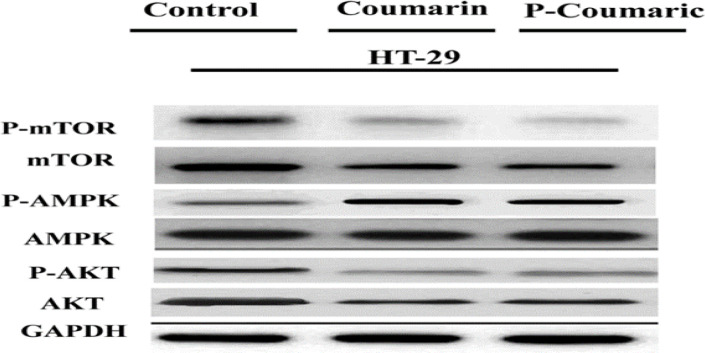
Determination of the effects of IC50 concentrations of coumarin and p-coumaric acid on the induction of autophagy and identification of mTOR, AMPK, AKT, Beclin-1, Atg5, and Lc3 proteins by Western blotting in HT-29 treated cells compared to control cells. As can be seen, the expression of P-mTOR and P-AKT was significantly decreased in the treated samples and the expression of P-AMPK, Beclin-1, Atg5, and Lc3 in the treated samples was increased compared to the control. GAPDH was also used as a loading control

Western blot examination was carried out to confirm the impacts of CO and PCA on the induction of autophagy and the proteins involved in this process. As shown in Figure 8, p-mTOR and P-AKT expressions were remarkably down-regulated in the groups treated with CO and PCA than the untreated group. Also, the expression of P-AMPK, *Beclin-1*, *Atg5*, and *Lc3* in the treated samples showed a significant rise compared to untreated cells. The data obtained from the study provide evidence that the mTOR/AKT/PI3K and Ampk/mTOR pathways are involved in the induction of autophagy by CO and PCA in HT-29 cancer cells. The mTOR protein is a negative regulator of autophagy, and the AMPK and AKT proteins act above mTOR. *Beclin-1*, *Atg5*, and *Lc3* are the genes involved in autophagy (Song et al., 2015 ▶).

## Discussion

Plants and their extracts are used in a wide range of phytomedicine with various natural functions. CO compounds have an important function in plant processes, including antioxidant, enzyme inhibitory, and regulation of the production of toxicants in various chemical reactions in the cell cycle (Kostova et al., 2005 ▶). CO is a herbal compound discovered in various plant species, including *Glycyrrhiza glabra*, *Lavandula angustifolia*, and *Cinnamomum zeylanicum* which has numerous healing effects (Song et al., 2015 ▶). Of all the diverse biological effects, anti-cancer actions, and their function in disorder prevention, the interest of investigators to analyze the properties of these compounds as cancer medicines has increased notably (Kawase et al., 2005 ▶).

This investigation desired to compare two herbal compounds of PCA and CO on HT-29 cells by inducing apoptosis and autophagy in fibrin hydrogel scaffolds. Herein, it was marked that both CO and PCA had concentration- and time-dependent toxic impacts on the viability of HT-29 cells further external distinctions, including transformations in nucleation, cell shrinkage, chromatin condensation by staining, and proving apoptosis. Compared to PCA, CO has shown significantly greater efficacy in inducing cell death, causing morphological changes, and impacting cell viability and gene assays. This confirms the potent effect of CO on cancer cells. At low concentrations of CO (IC50 25 μM), the highest cytotoxicity (reduced cell viability) was observed in colon cells. The toxic impact of both compounds was dose- and time-dependent, which ascended with growing quantity and cure time. After confirming the morphological observations, the results of qRT-PCR and western blot analyses also revealed an upregulation in the expression of pro-apoptotic and pro-autophagic genes and proteins, such as *Bax*, *p53*, *Atg5*, *Beclin-1*, *Lc3*, and *Bad*, as well as a downregulation in the expression of anti-apoptotic and anti-autophagic proteins like Bcl2, mTOR, and AKT, compared to the control group. This provides evidence of apoptotic and autophagic cell death in HT-29 cells following a 24-hour treatment with these compounds. They were remarkably more elevated than the untreated cells, confirming apoptosis and autophagy in the cells exposed to these compounds for 24 hr. Apoptotic regulators include anti-apoptotic *Bcl2* members, pro-apoptotic members (*Bax* and *Bad* genes), *caspases*, and *P53*. The Bax protein conducts the increase of cytochrome c and ultimately acts in the caspase cascade and apoptosis (Czabotar et al., 2014 ▶). mTOR is a PIK3 family kinase-dependent protein that controls and inhibits autophagy and blocks the phosphorylation of the ULK complex involved in autophagy initiation by binding to the complex. Therefore, the use of inhibitors of these proteins inhibits the function of mTOR and AKT, resulting in autophagy in cancerous tumors (Granville et al., 2007 ▶). The findings of this study align with previous research on the beneficial role of mTOR or AKT inhibition in cancer. This study exhibited that CO and PCA caused a reduction in the total status of mTOR and AKT proteins in HT-29 cells as well as the inhibition of their activation. It seems that CO and PCA may be able to induce AKT or mTOR degradation in HT-29 cells by arousing aggresome formation. 

Beclin-1 has a critical function in nucleation autophagy and the elongation of the spindle. Moreover, it's a multifunctional protein that interacts with Bcl2 or PI3K and has a critical function in regulating both autophagy and cell death. It has been reported that the upregulation of *Beclin-1* serves as a regulatory factor in the autophagy process in various types of cancer (Kang et al., 2011 ▶). For instance, variations in Beclin-1 gene expression can be studied in tumors of the ovary, breast, brain, liver, colon, and colorectal (Al-Shenawy et al., 2016 ▶). In this work, improved expression of *LC3* and *Atg5* genes in the treatment process confirmed the beginning of the autophagy pathway in HT-29 cancer cells. Since autophagy is a process with a dual nature that helps cells to survive and causes cell death by increasing stress. Here, to distinguish the dual nature of this process, the expression of AKT and AMPK proteins as important genes in the pathogenesis of malignancy have been suggested to enable the beginning of autophagy and the pathway of death induction.

In a study in 2018, Wang et al. examined the beginning of caspase-dependent cell death by a CO hybrid combination on lung cancer cells and explained that CO prevents the expression of the Bcl2 gene in the death pathway and triggered *Bax* gene expression. Therefore, in the cells that were exposed to CO, the presence of autophagosome was detectable. While eventually shown to be similar to most chemotherapy drugs, CO was able to activate the autophagy pathway in lung cancer cells (Wang et al., 2018 ▶). These results are compatible with the results of our study that autophagy pathway activator genes increased in the groups treated with CO and PCA compared to the control group. The new di- CO polysulfide compound synthesized by the researchers was able to block the cell cycle of colon cancer cells in the G2/M phase as a potent factor. The induction of apoptosis was also due to a reduction in *Bcl2* levels and an proliferation in *Bax* and *P53* levels (8-72 hr after treatment) in cells treated with this new compound (Saidu et al., 2012 ▶). Elinos-Baez et al. (2005) ▶ also investigated the effect of CO and hydroxy CO on *Bcl2* and *Bax* gene expression in human lung cancer cells and reached similar results. Both compounds resulted in reduced viability rate of the treated cells and altered their morphology (Elinos-Baez et al., 2005 ▶). Besides, multiple investigations have been conducted on the anti-cancer and cytotoxic impacts of CO and PCA, confirming our results, including that researchers found some natural CO compounds which induce autophagy in breast cancer (Ren et al., 2016 ▶) and prostate cancer (Suparji et al., 2016 ▶). The influence of the CO-synthesized product in an *in vivo* and *in vitro* investigation was considered. This syntactic combination had action against Ehrlich ascites carcinoma in mice and also showed a cytotoxic influence on human cancer cells MCF-7, HepG2, PC3, and HCT-116. The expression of the *P53* gene in mice treated with CO improved compared to the control group (Das et al., 2006 ▶). In the results of western blotting of the present study, CO and PCA were able to lower the inhibition of ATP-like proteins, such as mTOR and AKT in HT-29 cells. Yan et al. (2018) ▶ have demonstrated the shielding influence of glycocoumarine against acetaminophen-induced hepatotoxicity by enhancing the autophagy process and interfering with acetaminophen-induced oxidative stress in mouse hepatocytes (Yan et al., 2018 ▶).

PCA is a group of polyphenols derived from cinnamic acid whose effect on apoptosis and cell death in cancer cells has been studied extensively. For example, Ueda et al. (2019) ▶ in an investigation of the preservative impact of PCA against a copper-zinc superoxide dismutase 1 (neurotoxic) mutation in neurons, concluded that this compound activated the autophagy process and stopped the oxidative stress while being able to protect neurons against mutations. Western blot analysis also suggests that this compound induced the autophagy process by increasing the expression of the most famous autophagy marker LC3 and decreasing the P62 protein levels (Ueda et al., 2019 ▶). Researchers also confirmed the inhibitive impact of honey against colon cancer due to its high phenolic content, including PCA (Jaganathan et al., 2013 ▶). In an evaluation of the anti-tumor impact of PCA against lung cancer, this compound was able to induce mitochondrial apoptosis mechanism *in vivo* and *in vitro* by regulating *caspase-3*, *caspase-9*, *Bad*, *Bax*, and inhibiting *Bcl2*. It has led to cell death in lung cancer cells (Peng et al., 2015 ▶). Altogether, it could be suggested that PCA and CO can be considered suitable therapeutic candidates for the treatment of colon cancer due to their ability to activate cell death pathways, including apoptosis and autophagy. Further studies, particularly in the molecular context, are suggested, and of course the *in vivo* studying of these compounds on the animal can complement this work. 

We confirmed in this investigation that these compounds caused autophagy via PI3K/Akt/mTOR and AMPK/mTOR signaling pathways. A comparison of the impacts of the two compounds PCA and CO on colon cancer was conducted in which CO with a lower concentration (IC50=25μM) than PCA (IC50=150 μM) was capable to cause autophagy. Moreover, these two compounds have co-stimulated autophagy and apoptosis, thus significantly promoting cell death in cancer cells. On a final note, because CO is more potent in expressing apoptosis and autophagy inducer genes and proteins than in PCA in HT-29 cells, it can be suggested as a more effective drug in the treatment of colorectal cancer.

## Conflicts of interest

The authors have declared that there is no conflict of interest.
